# A common polymorphism in the retinoic acid pathway modifies adrenocortical carcinoma age-dependent incidence

**DOI:** 10.1038/s41416-020-0764-3

**Published:** 2020-03-09

**Authors:** Mirvat Surakhy, Marsha Wallace, Elisabeth Bond, Lukasz Filip Grochola, Husein Perez, Matteo Di Giovannantonio, Ping Zhang, David Malkin, Hannah Carter, Ivy Zortea S. Parise, Gerard Zambetti, Heloisa Komechen, Mariana M. Paraizo, Meghana S. Pagadala, Emilia M. Pinto, Enzo Lalli, Bonald C. Figueiredo, Gareth L. Bond

**Affiliations:** 1 Ludwig Institute for Cancer Research, Nuffield Department of Clinical Medicine, University of Oxford, Oxford, UK; 2 Institute for Regenerative Medicine (IREM), University of Zurich, Zurich, Switzerland; 3 Department of Surgery, Cantonal Hospital Winterthur, Winterthur, Switzerland; 4 Faculty of Technology, Design and Environment, Oxford Brookes University, Oxford, UK; 5 Division of Hematology/Oncology, The Hospital for Sick Children, Department of Pediatrics, University of Toronto, Toronto, Canada; 6 Division of Medical Genetics, Department of Medicine, University of California, San Diego, USA; 7 Instituto de Pesquisa Pelé Pequeno Príncipe, Faculdades Pequeno Príncipe, Curitiba, Brazil; 8 Department of Pathology, St. Jude Children’s Research Hospital, Memphis, TN USA; 9 Institut de Pharmacologie Moléculaire et Cellulaire CNRS, Université Côte D’Azur, Inserm, Valbonne, France; 10 Departamento de Saúde Coletiva, Universidade Federal do Paraná, Curitiba, PR Brazil; 11 Centro de Genética Molecular e Pesquisa do Câncer em Crianças (CEGEMPAC), Curitiba, PR Brazil

**Keywords:** Gene expression, Paediatric cancer

## Abstract

**Background:**

Genome-wide association studies (GWASs) have enriched the fields of genomics and drug development. Adrenocortical carcinoma (ACC) is a rare cancer with a bimodal age distribution and inadequate treatment options. Paediatric ACC is frequently associated with *TP53* mutations, with particularly high incidence in Southern Brazil due to the *TP53* p.R337H (R337H) germline mutation. The heterogeneous risk among carriers suggests other genetic modifiers could exist.

**Methods:**

We analysed clinical, genotype and gene expression data derived from paediatric ACC, R337H carriers, and adult ACC patients. We restricted our analyses to single nucleotide polymorphisms (SNPs) previously identified in GWASs to associate with disease or human traits.

**Results:**

A SNP, rs971074, in the alcohol dehydrogenase 7 gene significantly and reproducibly associated with allelic differences in ACC age-of-onset in both cohorts. Patients homozygous for the minor allele were diagnosed up to 16 years earlier. This SNP resides in a gene involved in the retinoic acid (RA) pathway and patients with differing levels of RA pathway gene expression in their tumours associate with differential ACC progression.

**Conclusions:**

These results identify a novel genetic component to ACC development that resides in the retinoic acid pathway, thereby informing strategies to develop management, preventive and therapeutic treatments for ACC.

## Background

Adrenocortical carcinoma (ACC) is a rare cancer that usually occurs in the first or after the fifth decade of life.^1^ The incidence of most paediatric carcinomas increases progressively with age, but ACC has a peak incidence between birth and 4 years of age.^2^ Li-Fraumeni syndrome (LFS) families have a higher risk of developing paediatric ACC, with more than 50% of all children with ACC having a heritable *TP53* mutation.^1^ The germline R337H mutation was spread through a founder effect.^3,4^ Newborn screening for the R337H in Southern and South-eastern Brazil showed that this mutation has a frequency of 0.21–0.30%.^5–7^ This mutation is responsible for the increased incidence of paediatric ACC in Paraná state (3.4 per million before the age 15 years per year compared to 0.3 per million in the USA, or 6.4/million children younger than 10 years per year).^2,5^ A hospital-based cohort estimated that the penetrance of this mutation for paediatric ACC is 10%.^8^ However in a large population-based cohort, the penetrance for paediatric ACC is less than 5%, whilst the overall cancer risk among females and males is less than 50% before the seventh decade.^5,7^ The frequency of R337H prevalence among paediatric ACC varies from 75–96%, and is nearly 15% among adult ACC in Southern and South-eastern Brazil.^7,9,10^ The phenotypic heterogeneity among R337H carriers in terms of cancer risk and the variability in age-of-onset may indicate the involvement of environmental and/or genetic variants that are able to modulate cancer development.^5,7^ The enormous heterogeneity in cancer development noted in families carrying the germline R337H mutation^5^ has prompted us to gain further insight into potential genetic factors cooperating to modify the age-dependent incidence of cancer onset in the adrenal cortex.

ACC requires more effective treatment strategies. The prognosis of children with residual or metastatic ACC is dismal^11^ and can be worse in older children^12^ and adult patients.^13,14^ Knowing which gene(s) play important roles in the multistep tumorigenesis process in adrenocortical cells will certainly inform strategies to develop preventive and/or therapeutic protocols. Growing genomic data has allowed recent studies to highlight the importance of heritable genetic determinants in identifying potential drug targets and disease treatments.^15–17^ For example, one analysis showed that if a drug target has heritable genetic data supporting its role in a given disease, the chance of successful clinical development of a drug is doubled.^16^ Through advances in genome genotyping and sequencing technologies, the identification of genetic variations associating with disease traits has rapidly increased. For example, GWASs have identified over 900 SNPs significantly associated with cancer susceptibility traits.^18^ However, the necessity of large cohort sizes in GWASs to overcome the burden of multiple hypothesis testing has resulted in a relative deficit in our knowledge of the genetic variants underlying rare cancers like ACC.^19,20^

The genetic data supporting the role of p53 in ACC has improved the early diagnosis of ACC in *TP53* mutation carriers through asymptomatic screening.^5,21^ However, targeting p53 in either the preventative or therapeutic setting remains a challenge.^1^ The identification of other more druggable gene targets with genetic associations with ACC could prove useful, whereby SNPs in genes that associate with differential ACC cancer risk in *TP53* mutation carriers could help to identify novel druggable genes and/or pathways. SNPs in the p53 pathway have been clearly shown to associate with differential cancer risk. Indeed, SNPs in well-described p53 pathway genes have been frequently identified in GWASs of seemingly sporadic cancers.^18^ In fact, one p53 pathway SNP, MDM2 SNP309 that associates with differential expression levels of a key regulator of p53, has been shown to modify cancer risk in *TP53* mutation carriers with LFS.^22^ Specifically, LFS families with more penetrant *TP53* mutations relative to the Brazilian R337H, are at higher risks of developing many types of cancer, such as sarcomas, breast, and brain cancers, as well as ACC. MDM2 SNP309 has been shown to modify this cancer risk in multiple cohorts of *TP53* mutation carriers in an age- and gender-dependent manner.^22–26^ Importantly, genetically engineered mouse models carrying either alleles of *MDM2* SNP309 and a highly penetrant LFS p53 mutation demonstrated similar allele-specific differences in age-dependent cancer incidence.^26^ Together, observations such as these suggest that SNPs could potentially modify ACC cancer risk. Here, we studied two independent cohorts of ACC patients (paediatric and adult) to demonstrate that a SNP in a druggable gene and pathway significantly associates with ACC age-dependent incidence.

## Methods

### Study cohorts

#### *TP53* p.R337H paediatric ACC cohorts

A cohort of children diagnosed with ACC with the R337H germline mutation was used in this study. We divided this cohort into a discovery cohort (BRZ1, *N* = 26) and a validation cohort (BRZ2, *N* = 16) based on when the DNAs were received from the Pathology unit. The patients were treated in the Pequeno Príncipe Hospital, Brazil. This study was approved by the Ethics Committee from Pequeno Príncipe Hospital, and authorisation was obtained by signing a consent form. Clinical data that included the age at diagnosis for each patient, gender, and *TP53* mutation status were available.

#### Adult ACC cohort

A retrospective database for primarily sporadic ACC patients (*N* = 92) was analysed from the Cancer Genome Atlas.^27^ These patients were diagnosed with adult ACC and blood-derived genotype data from the Affymetrix™ Genome-Wide Human SNP Array 6.0 was available together with the relevant clinical information. Approval from the National Institute of Health (NIH) Data Access Committee was obtained (request number 34594–5). Somatic mutations of *TP53* for this data set were retrieved from cBioPortal (http://www.cbioportal.org/) website (accessed December 2018).

### Genomic DNA preparation

Genomic DNAs from the BRZ patients were extracted from peripheral blood using the ReliaPrep™ gDNA kit as per the manufacturer’s recommendations (A2051, Promega).

### SNP genotyping

Genomic DNAs from the BRZ cohort were genotyped using the Infinium^©^ LCG assay on the Human Omni 2.5–8 v1.3 BeadChip (Illumina platform), according to the manufacturer’s instructions and at the High-Throughput Genomics at the Wellcome Trust Centre for Human Genetics, University of Oxford.

### Cancer GWAS and GWAS SNPs

The GWAS Catalog was downloaded from the European Bioinformatics Institute (EMBL-EBI) website (https://www.ebi.ac.uk/gwas/) on February 2018. We found 35,705 GWAS lead SNPs in studies with individuals with European (EUR) ancestry. We then retrieved all closely linked SNPs (proxies) based on the 1000 Genomes phase 3 data acquired through the web server: rAggr (http://raggr.usc.edu), and only considered the proxies that meet the following criteria: population: EUR; minimum MAF (minor allele frequency): ≥0.01; *r*^2^ ≥ 0.8; maximum distance: 500 kb; maximum # Mendel error: 1; Hardy-Weinberg equilibrium (HWE) *p*-value: 1e-6; and minimum genotyping rate of 95%. Cancer GWAS proxy SNPs and GWAS proxy SNPs were then pulled to intersect with the significant SNPs genotyped on the Infinium array as described below.

SNPs on the Infinium genotyping array with MAF ≥ 0.05 were selected based on dbSNP150 hg19 genome build. Briefly, snp150Common.txt were accessed in August 2018 from http://hgdownload.soe.ucsc.edu/goldenPath/hg19/database/. Triallelic SNPs were removed. SNPs with MAF ≥ 0.05 (8,209,080 SNPs) were then pulled to identify the number of SNPs genotyped on the Infinium array with MAF ≥ 0.05 among cancer GWAS and GWAS SNP lists.

### SNP analyses

The following methods were used to analyse the BRZ cohort. First, SNPs mapped to chromosome: 0 (unknown), XY (pseudoautosomal), Y, or MT (mitochondria) and SNPs mapped to chromosome position 0 were removed. Only females were considered for the SNPs on the X Chromosome. A MAF threshold was set at 0.05 (SNPs with MAF < 0.05 were removed). Nonpolymorphic SNPs and duplicated SNPs (SNPs with two rsIDs at the same position) and SNPs that failed genotyping for all patients were removed. For each SNP, heterozygous and homozygous minor allele genotype calls were combined into the same group for statistical analysis, to overcome the missing genotypes and/or low patient numbers per genotype within these two small cohort sizes. A Log-ranked non-parametric test, the Mann–Whitney *U* test, two-sided, was used to determine statistical differences between genotype and age-of-tumour onset. The analysis was performed in base R using the Wilcoxon test. SNPs in the BRZ1 cohort with a raw *p*-value < 0.05 in the Mann–Whitney *U* test were then likewise tested for significance in the BRZ2 cohort and examined for consistent effects. The subset of SNPs having a raw *p*-value < 0.05 in the Mann–Whitney *U* test in both cohorts and showing a consistent effect were considered for further examination and validation in the adult ACC cohort.

For the primarily adult ACC cohort, reference SNPs rsIDs were extracted from the Affymetrix Genome-Wide Human SNP 6.0 Array names according to https://www.ncbi.nlm.nih.gov/geo/query/acc.cgi?acc=%20GPL6801 (accessed July 2018). The primarily adult ACC cohort genotyping was on the Affymetrix SNP array, while the paediatric BRZ cohorts were genotyped using the Infinium LCG array. Thus, when testing significant SNPs (raw *p* < 0.05) identified in both BRZ cohorts, we either utilised the genotypes for the same SNP, if directly genotyped SNP on the Affymetrix SNP array, or we utilised the genotypes of a closely linked proxy SNP genotyped on the Affymetrix SNP array. Proxy SNPs were determined using rAggr (http://raggr.usc.edu) for the EUR population. If multiple proxies for a given significant SNP were genotyped in the adult ACC cohort, the most closely linked SNP was utilised. The heterozygous and homozygous minor allele genotypes were combined for statistical analyses if the number of minor homozygotes were ≤4. A Log-ranked non-parametric test, two-sided, the Mann–Whitney *U* test (2 groups) or the Kruskal–Wallis test (three groups), was applied to determine statistical differences between genotype and age of tumour onset. P-values were subsequently adjusted for multiple hypothesis correction (Bonferroni correction).

### mRNA expression levels and survival analyses

RNA-seq data for the adult ACC cohort (illumine hiseq Level 3 RSEM normalised genes) were retrieved from http://firebrowse.org/?cohort=ACC accessed February 2019. Genes involved in the regulation of retinoid metabolism and those involved in retinoic acid receptor-mediated signalling were retrieved from http://software.broadinstitute.org/gsea/msigdb/cards/GO_RETINOIC_ACID_METABOLIC_PROCESS.html (GO:0042573) and http://software.broadinstitute.org/gsea/msigdb/cards/PID_RETINOIC_ACID_PATHWAY.html, accessed 18 July 2019. The cut-off point between high and low mRNA expression levels for each gene was computed based on the maximally selected rank statistics using the “survminer” package in R. mRNA levels with values above the cut-off point were considered high and below were considered low.

Survival data for the adult ACC were retrieved from Liu et al.,^28^ Table S1 Tab TCGA-CDR, accessed May 2019. The progression-free interval (PFI) endpoint was analysed (PFI, the length of time during and after the treatment of a disease when a patient lives with the disease but it does not get worse). The patients in the adult ACC cohort were randomly separated into Discovery and Validation cohort pairs at 50:50 ratios 10 times using the “R Base” package.^29^ We first performed Kaplan–Meier survival analyses to estimate the survival probability as a function of time in relation to mRNA expression levels for each transcript (patients with low versus high expression levels in tumours). The Log-Mantel and Haenszel survival test (a Log-rank test) was used to determine the statistical differences betwen the groups. Genes that were significant with *p* < 0.05 were tested in the paired Validation cohort. In the Validation cohort the analyses were undertaken in the exact same manner except for the final adjustment of the p-values for multiple hypothesis testing, which was performed using the Bonferroni method. Adjusted *p* < 0.05 were considered significant. These analyses were performed in each of the 10 Discovery/Validation pairs and those genes whose transcript levels associated with PFI six times or more were considered hits.

Gene expression data from two independent paediatric ACC cohorts were used in this study: the Children’s Oncology Group (COG, *n* = 34, accession number; GSE76019) and International Pediatric Adrenocortical Tumor Registry group (IPACTR, *n* = 19, accession number; GSE76021). The data from the COG and the IPACTR were accessed via the Gene Expression Omnibus (GEO) in June 2019. For these cohorts, the progression-free survival (PFS) endpoint was considered (PFS, the time elapsed between protocol enrolment and the date of disease progression, death, or most recent follow-up).^30^ mRNA expression data was available as a normalised log2 signal. The data were generated on two different array platforms: IPACTR on the GLP96 platform, COG on the GPL13158 platform. In order to maximise consistency in the analyses, we considered only probes with the same Transcript ID design. The cut-off point between high and low mRNA expression levels, the statistical difference in survival, and the approach used to validate the significant genes were performed as described above.

All analyses were performed on R (version 3.5.1) using the following packages “data.table”, “dplyr”, “reshape2”, “tidyr”, “plyr”, “survminer” and “survival” in addition to base R. Plots were generated using Prism 7 (GraphPad) and R using the “qqman”, “gplots” and “ggplot2” packages. All statistical tests were two-sided except where otherwise stated. Pathway diagram was created with BioRender.com.

## Results

In order to identify potential genetic modifiers underlying ACC development that could improve cancer risk management and treatment, we genotyped a total of 42 *TP53* R337H carriers with ACC, divided into a discovery cohort (BRZ1, *N* = 26) and a validation cohort (BRZ2, *N* = 16). All BRZ samples had a >98% genotype call rate, with an average overall call rate of 99.6%. The clinical characteristics of the paediatric patients of both cohorts are shown in Supplementary Table 1. All children (29 girls and 13 boys) were carriers of the R337H mutation and were diagnosed at ages ranging from 0.14 to 14.7 years. Family history showed that three children were from LFS families, 17 children were from Li-Fraumeni-Like (LFL) families and 22 with no LFS/LFL family histories (Supplementary Table 1) according to the most stringent criteria.^31^

### Cancer GWAS SNPs and ACC age-of-onset in R337H mutation carries

**Fig. 1:**
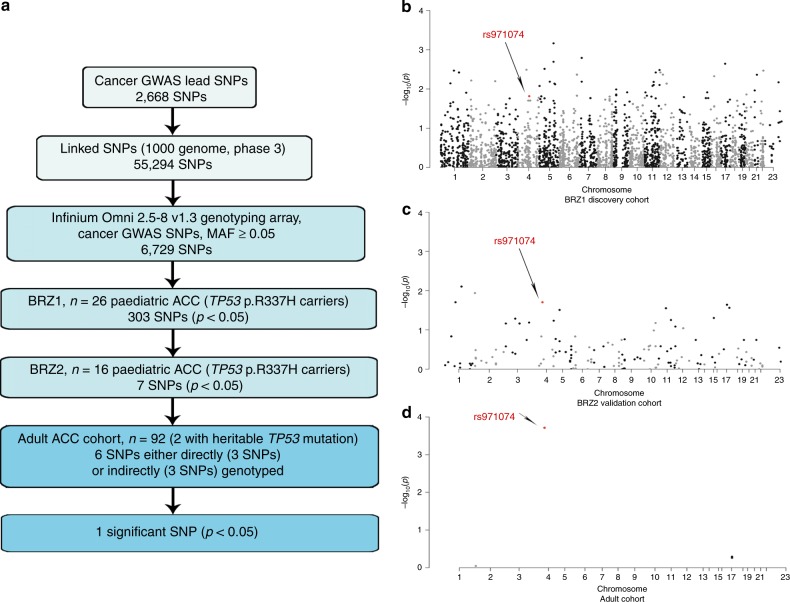
Cancer GWAS approach to identify the genetic modifiers in ACC age-of-onset. **a** A flow chart representing the focused analytical approach taken to identify potential cancer GWAS SNPs which associate with differential age of ACC onset. **b–d** Manhattan plot representing cancer GWAS SNPs tested in BRZ1 (discovery) cohort with p-value represented in −log_10_(*p*) for each SNP (n = 6059) (**b**). Manhattan plot representing the significant cancer GWAS SNPs (*p* < 0.05) in BRZ1 and their validation in the BRZ2 cohort (*n* = 282, out of 303 significant in BRZ1) (**c**). Manhattan plot representing significant the cancer GWAS SNPs in both BRZ1 and BRZ2 and validated in the adult ACC cohort (*n* = 6, out of 7 significant in BRZ1 and BRZ2) (**d**). The significant SNP in the 3 cohorts is marked in red and pointed with an arrow.

The small cohort size and narrow age-of-onset window necessitated a focused analytical approach in the search for SNP modifiers. Therefore, we focused our search on SNPs that have been previously found to associate with differential cancer risk in GWAS (Fig. 1a). This process began by procuring the 2,668 GWAS lead SNPs in the GWAS catalog found to significantly associate with cancer risk among Europeans. In total, these SNPs were found to associate with differential risk of 33 different cancers. Next, proxy SNPs linked to these cancer GWAS tags were obtained. SNP linkage information is not available for the Brazilian population, but it is thought that the R337H mutation originates from a founder effect of European origin.^3,4^ Therefore, we retrieved proxies that are linked in the EUR. This resulted in 55,294 cancer GWAS SNPs. Of the cancer GWAS SNPs, 6,729 SNPs were on the Infinium genotyping array with MAF ≥ 0.05. 303 cancer GWAS SNPs showed significant allelic differences with ACC age-of-onset in BRZ1 (*p* < 0.05, Mann–Whitney *U* test). 282 SNPs were tested in BRZ2 (MAF ≥ 0.05 in BRZ2), of these, seven SNPs were also associated significantly (*p* < 0.05, Mann–Whitney *U* test) with differential age-of-onset in a consistent manner whereby the same allele associated with an earlier diagnosis (Fig. 1a).

### Cancer GWAS SNPs and ACC age-of-onset in sporadic adult ACC

**Fig. 2:**
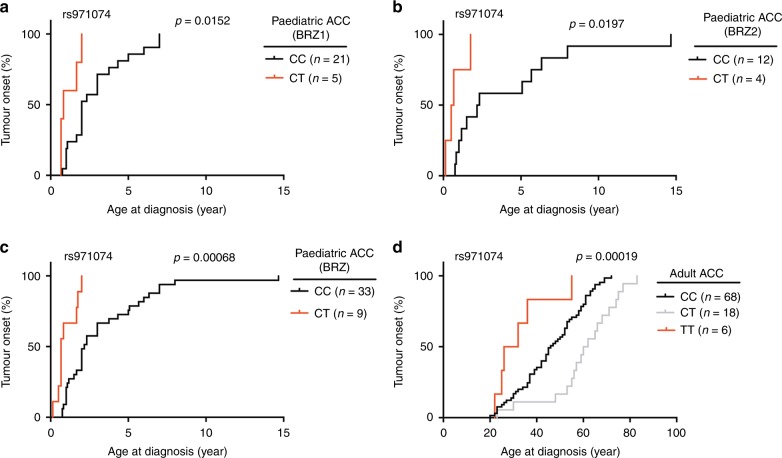
rs971074 SNP modifies the age of ACC onset. Kaplan–Meier plots representing the age of tumour onset for both the major allele homozygous (CC) and the minor allele heterozygous (TC) and homozygous (TT). In **a** the ages of onset of the first paediatric ACC cohort is plotted (BRZ1) (*p* = 0.0152, two-sided Mann–Whitney *U test*). In **b** the second paediatric cohort is plotted (BRZ2) (*p* = 0.0197, two-sided Mann–Whitney *U* test). In **c** the paediatric cohort combined (BRZ) is plotted (*p* = 0.00063, two-sided Mann–Whitney *U* test). In **d** the patients in the primarily adult ACC cohort are plotted (*n* = 92), *p* = 0.00019, Kruskal–Wallis test.

The cohort of primarily adult ACC patients included 92 individuals (60 women and 32 men) diagnosed with a mean age of 47.16 years (range 14–83 years). Two patients were carriers of the R337H germline mutation (diagnosed at ages 23 and 30 years). For all patients, genotyping information was available from blood DNA (Affymetrix SNP 6.0 Array), while somatic mutation data from the tumours was available for 91 patients (Supplementary Table 2). Out of the seven SNPs that significantly associated with differential age-of-onset of paediatric ACC, six SNPs could be studied in the primarily adult ACC cohort, as three were successfully genotyped with the Affymetrix array, and three SNPs had successfully genotyped proxies. One of these six SNPs, rs971074, significantly associated with differential age-of-onset (adj. *p* = 0.00114, Kruskal–Wallis test). rs971074 (C/T, chr4:100341861, Genome Reference Consortium Human Build 37 (GRCh37)/hg19) is a synonymous SNP located in exon 6 of the alcohol dehydrogenase 7 gene (*ADH7)* gene (Fig. 1b-d). The *TP53* mutation carriers with rs971074 minor allele (CT) were diagnosed at a median age of 0.85 year in the BRZ1 and at a median of 0.585 year in BRZ2 cohort. However, carriers of the homozygous major allele (CC) were diagnosed at a median age of 2 years (BRZ1, *p* = 0.0153, Mann–Whitney *U* test), and at a median of 2.25 years (BRZ2, *p* = 0.0197, Mann–Whitney *U* test) Table 1, Fig. 2a, b. When the patients of BRZ1 and BRZ2 are combined (BRZ), CT patients were diagnosed at a median age of 0.67 years compared to a median age of 2.17 years for CC patients (*p* = 0.00068, Mann–Whitney *U* test) Table 1 and Fig. 2c.

**Table 1:** rs971074 in the paediatric BRZ ACC cohort.

Cohort			Patient numbers	Mean (years)	Median (years)
BRZ1	Genotype	Major (CC)	21	2.9	2
		Minor (CT)	5	1.168	0.83
	MAF		0.0962		
	*p*-value (MWU)		0.0153		
BRZ2	Genotype	Major (CC)	12	4.13	2.25
		Minor (CT)	4	0.765	0.585
	MAF		0.125		
	*p*-value (MWU)		0.0197		
BRZ	Genotype	Major (CC)	33	3.345	2.17
		Minor (CT)	9	0.999	0.67
	MAF		0.1071		
	*p*-value (MWU)		0.000068		

*MAF* minor allele frequency, *MWU* Mann–Whitney *U* test.

In the primarily adult ACC cohort, CC patients were diagnosed 16 years earlier than TT patients (at a median age of 45 and 29 years, respectively. *p* = 0.0498, Mann–Whitney *U* test) Table 2 and Fig. 2d, in a manner similar to the paediatric cohort. In contrast to the paediatric cohort, patients heterozygous for rs971074 were diagnosed at older ages (16 years later than CC patients, *p* = 0.00038; 32 years later than TT patients, *p* = 0.00414, Mann–Whitney *U* test).

**Table 2:** rs971074 in the adult ACC cohort.

Cohort			Patient number	Mean (years)	Median (years)
Adult ACC	Genotype	CC^a^	68	45.01	45
		CT^b^	18	60.1	61
		TT^c^	6	32.67	29
	MAF		0.163		
	*p*-value (KW)		0.00019		
	adjusted *p*-value (Bonferroni)		0.00114		
	*p*-value (MWU) (CC vs TT)		0.0498		
	*p*-value (MWU) (CC vs CT)		0.00038		
	*p*-value (MWU) (TT vs CT)		0.0041		
Adult ACC (somatic wild type p53)	Genotype	CC	50	45.34	46.5
		CT	16	60.19	61
		TT	5	32	26
	MAF		0.1831		
	*p*-value (KW)		0.00075		

*MAF* minor allele frequency, *KW* Kruskal–Wallis, *MWU* Mann–Whitney *U* test.

^a^CC: Homozygous major.

^b^CT: Heterozygous.

^c^Homozygous minor.

Altogether, these data show that the rs971074 SNP associates with differential ACC age-of-onset in both *TP53* mutation carriers and non-carriers alike. However, in ACC patients with no inherited *TP53* mutations, the *TP53* gene is known to be somatically mutated (19.6%, cBioPortal). In the adult cohort, 18 patients (19.6%) had known pathogenic *TP53* mutations in their tumours, and 2 had the R337H germline mutation. When we restrict our analyses to those 71 patients without either inherited or somatic *TP53* mutations, and excluding the patient with no somatic mutation data, similar allelic differences in the age-of-onset were found (*p* = 0.00075, Kruskal–Wallis test, Table 2 and Supplementary Fig. 1), wherein TT patients were diagnosed at a median age of 26 years compared to a median age of 46.5 years for the CC patients. This corresponds to a 20.5 year earlier diagnosis for TT patients demonstrating the apparent *TP53* mutation-independence of these associations.

It is important to note that in a smaller paediatric ACC cohort (*N* = 21, The Hospital for Sick Children, Canada) of *TP53* mutation carriers with more penetrant mutations and diagnosed at ages ranging from 1 to 17.83 years (Supplementary Table 3), no significant difference in ACC age-of-onset with the rs971074 SNP genotype was observed (Supplementary Table 4).

### GWAS SNPs and ACC age-of-onset

**Fig. 3:**
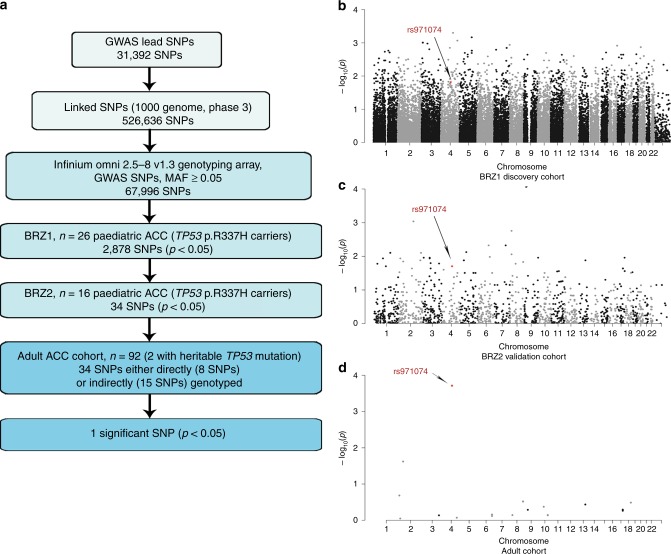
GWAS approach to identify the genetic modifiers in ACC age-of-onset. **a** A flow chart representing the focused analytical approach taken to identify potential GWAS SNPs, which associate with differential age of ACC onset. **b–d** Manhattan plot representing GWAS SNPs tested in BRZ1 (discovery) cohort with p-value represented in −log_10_(p) for each SNP (*n* = 61,609) (**b**). Manhattan plot representing the significant cancer GWAS SNPs (*p* < 0.05) in BRZ1 and their validation in the BRZ2 cohort (*n* = 2,644, out of 2,878 significant in BRZ1) (**c**). Manhattan plot representing the significant cancer GWAS SNPs in both BRZ1, BRZ2 and validated in the adult ACC cohort (*n* = 23, out of 34 significant in BRZ1 and BRZ2) (**d**). The significant SNP in the three cohorts is marked in red and pointed with an arrow.

The rs971074 SNP was included in our list of cancer GWAS SNPs due to its association with differential cancer risk in the upper aerodigestive tract, particularly oesophageal cancers.^32,33^ To further explore this association in ACC, we included GWAS SNPs known to associate not only with cancer, but also with all other diseases and traits that have been studied in GWAS. Specifically, in total, 31,392 lead SNPs can be found in the GWAS catalog. For these, we identified 526,636 closely linked proxy SNPs. Of these, 67,996 SNPs with MAF ≥ 0.05 were present on the genotyping array. Among these, 2,878 SNPs were associated with the ACC age-of-onset in BRZ1 (*p* < 0.05, Mann–Whitney *U* test). 2,644 SNPs were tested in BRZ2 (MAF ≥ 0.05), of these, 34 SNPs were also found to be associated with age-of-onset in a consistent manner (*p* < 0.05, Mann–Whitney *U* test). In the adult ACC cohort, we were able to explore potential associations with differential age-of-onset for 23 of the 34 SNPs (8 SNPs directly and 15 SNPs through proxies) Fig. 3a–d. Interestingly, only the rs971074 SNP was significant after multiple hypothesis correction (adj. *p* = 0.0044, Kruskal–Wallis test), thereby underlining the importance of the GWAS SNP’s association with cancer and not other diseases and traits.

### Expression levels of RA pathway genes in ACC associate with tumour progression

**Fig. 4:**
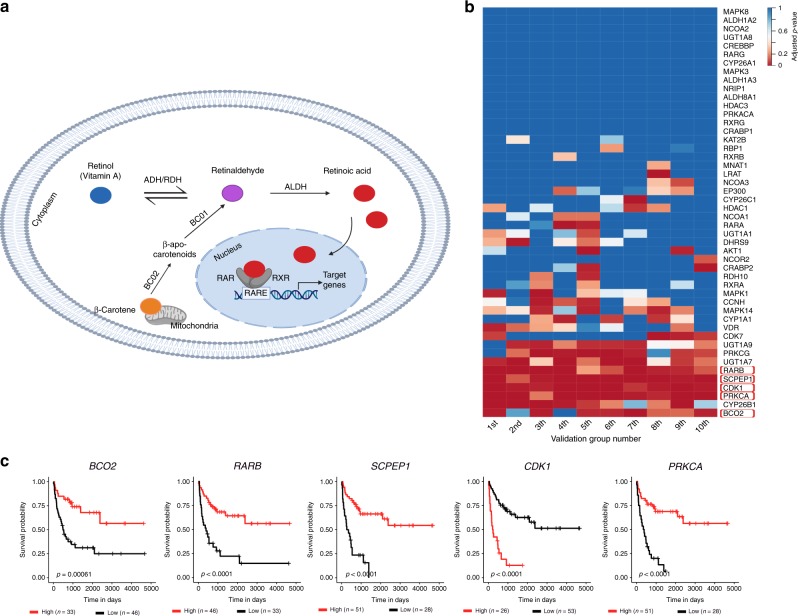
Expression levels of RA pathway genes in ACC associate with progression in adult patients. **a** A depiction of the RA signalling pathway. Retinol (Vitamin A) is reversibly oxidised to retinaldehyde by the alcohol dehydrogenase (ADH) or retinol dehydrogenase (RDH) enzymes. Retinaldehyde is irreversibly oxidised to retinoic acid (RA), the active form of retinol, by the action of aldehyde dehydrogenases (ALDH) family members, also known as retinaldehyde dehydrogenases (RALDH). Retinaldehyde can also be produced from β-carotene. Beta-carotene oxygenase 2 (BCO2) asymmetrically cleaves β-carotene to generate β-apo-10′-carotenal, which is then converted to retinaldehyde, by BCO1. RA is the ligand for the nuclear receptors, retinoic acid receptor (RAR) and retinoid X receptor (RXR). RA regulates the transcription of its target genes following its binding to the RAR/RXR- retinoic acid responsive element (RARE) complex. **b** A heatmap representing the level of significance of the association of the transcript levels of RA pathway genes with progression-free interval of ACC patients. The results for the transcripts for all 48 genes are presented for the 10 Validation groups. *P*-values represent the Bonferroni adjusted *p*-values and considered significant if *p* < 0.05. Genes were considered hits if they were found to be significantly associated with ACC in six or more of the Validation groups and are boxed in red. **c** Kaplan–Meier survival curves for the significant hits. The plots were derived using all adult ACC patients (*n* = 79). Statistical analyses were performed using the Log-rank test and the p-values are indicated on the graphs.

ADH7 is involved in the production of RA from retinol (also known as vitamin A), which can induce differentiation and inhibit cellular growth (Fig. 4a). This pathway is therapeutically malleable and RA isomers are utilised in both cancer prevention and treatment strategies.^34,35^ Our data thus far suggest that the RA pathway could be important in ACC. To further test this hypothesis, we explored the association of RA pathway gene expression with ACC progression and survival. We focused our analyses on 50 RA pathway genes, whereby 30 genes are known to be involved in RA signalling and 20 genes in RA metabolism. In the adult ACC cohort, we processed RNA-seq data derived from tumours. We were able to detect significant RNA levels of 48 of the 50 genes. Next, we randomly assigned the patients into Discovery and Validation groups at a 50:50 ratio. We repeated these analyses ten times. For each Discovery group, we first separated the patients into two groups based on the expression levels of a given RA pathway gene using a Rank statistic (high and low expressing groups). We then explored potential differences in PFI between these two groups using a Log-rank test. In the patients of the Discovery cohorts, we identified an average of 28 transcripts (ranging from 20 to 33) that were associated with PFI with a raw *p* < 0.05. Next, we sought validation of the associations identified in the Discovery groups, in the paired Validation groups. In the Validation groups, the raw-p-values for each association were adjusted for multiple hypothesis testing and significance was considered with an adjusted *p* < 0.05 (Bonferroni adjustment, Supplementary Table 5). Interestingly, expression levels of 5 different genes consistently associated with PFI in minimally 6 different Discovery/Validation cohort pairs (Fig. 4b). One of the genes (*BCO2)* is involved in RA metabolism, while the other 4 genes (*RARB*, *SCPEP1*, *CDK1* and *PRKCA*) are involved in RA signalling (Fig. 4c).

**Fig. 5:**
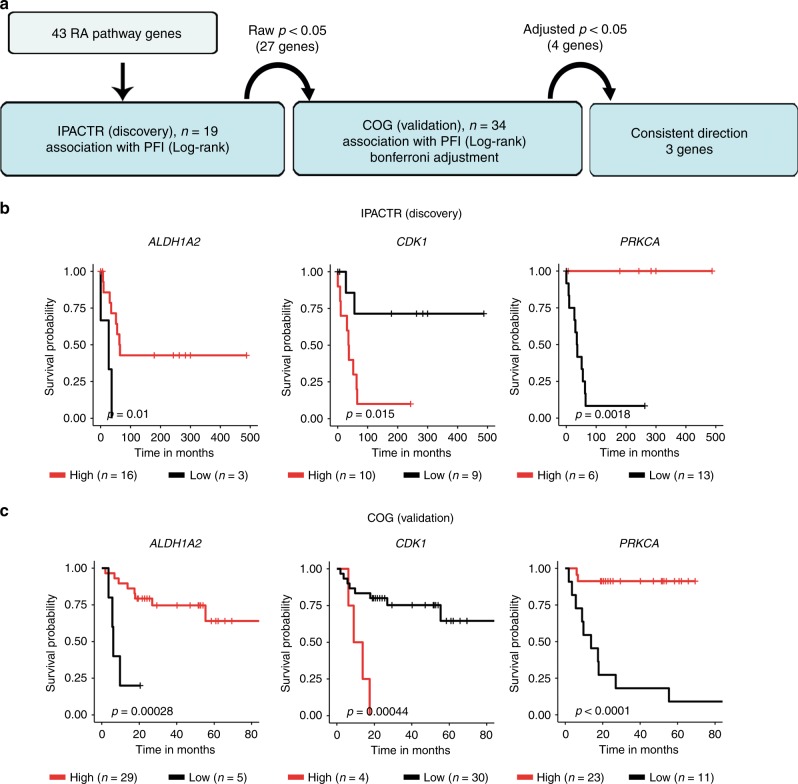
Expression levels of RA pathway genes in ACC associate with progression in paediatric patients. **a** A flow chart representing the analytical approach taken to identify the RA pathway genes that are associated with paediatric ACC progression. 17 genes showed an association with PFS in the Discovery cohort (Log-rank test, raw *p* < 0.05), among these 17 genes, 3 genes showed a significant association with PFS in the Validation cohort (Log-rank test, Bonferroni adjusted *p* < 0.05) in consistent manner. **b**, **c** Kaplan–Meier survival curves showing the RA pathway genes that are associated with PFS in paediatric ACC patients. Statistical analysis was performed using the Log-rank test and p-values are indicated on the graphs.

These data implicate a role for RA pathway genes in ACC progression. To further test this hypothesis, we next explored similar associations in paediatric ACC. We utilised two independent paediatric ACC cohorts with microarray gene expression data associated with outcome information (IPACTR and COG).^30^ We investigated the expression data from histologically confirmed ACC paediatric patients (19 from the IPACTR and 34 from the COG).^30^ We analysed these cohorts in a similar manner as with the adult ACC cohort, and thus randomly assigned the IPACTR as the Discovery cohort and the COG as the Validation cohort, and explored potential associations of gene expression for the 50 RA pathway genes with PFS using a Log-rank test. Specifically, as the cohorts utilised different arrays, we restricted our analyses to shared probes to maximise our ability to draw comparisons. Out of the 50 RA pathway genes, we were able to identify 79 shared probes for 43 RA pathway genes. Using these data and the above-described analytical pipeline, we determined that the expression levels of 4 different transcripts from three different genes associated with PFS in both cohorts: Discovery cohort: raw *p* < 0.05; Validation cohort: adjusted *p* < 0.05. One of the genes (*ALDH1A2*) is involved in RA metabolism, while the other two genes (*CDK1*, and *PRKCA*) are involved in RA signalling (*p* < 0.05, Bonferroni adjustment, Fig. 5a–c, Supplementary Table 6).

## Discussion

In this report, we explored whether SNPs could potentially modify ACC cancer risk and thus give us a better understanding of ACC development and possible indications of novel treatments. Due to the fact that this is a rare cancer, we undertook a focused analytical approach, whereby we restricted our analyses to SNPs previously identified in GWAS studies to associate with disease or other human traits. We utilised two independent cohorts of ACC patients and clearly demonstrated that a SNP (rs971074 in *ADH7*) in a druggable pathway significantly associated with the age of ACC onset, suggesting an increased risk associated with ACC age-of-onset. We demonstrated that the minor allele of rs971074 in *ADH7* associates with a significantly earlier age of paediatric ACC in R337H carriers (CT) and adult ACC (TT), relative to patients homozygous for the major allele (CC) in a manner that is seemingly independent of both inherited and somatic *TP53* mutations. It is important to note that although the associations with the age-of-onset for the different genotypes were significant in both the paediatric and adult ACC cohorts, there are some differences. For example, in the adult cohort, the heterozygous patients (CT) demonstrated the latest average age-of-onset, while in the paediatric, the heterozygotes were the youngest. It will be important to validate the associations in a second adult cohort, but this could further reflect the known genetic differences in the development of paediatric and adult ACC.^36,37^ For instance, amplification of chromosome 9q occurs in 90% of paediatric ACC tumours but not in adult tumours.^36^ Moreover, although upregulation of the Wnt/β-catenin signalling is common in paediatric and adult ACC, mutations in *ZNRF3* are only observed in adult tumours.^36,37^

We went on to demonstrate that amongst all SNPs identified to associate with human diseases or traits, only this SNP significantly and reproducibly associated with ACC age-of-onset. Intriguingly, this SNP was shown to associate in GWAS with differential risk for the development of cancer, namely with upper aerodigestive tract cancer risk.^32,33^ The rs971074 SNP is a synonymous SNP found in exon 6 of *ADH7*. ADH7 is important in retinoic acid (RA) signalling as it is crucial for the production of RA from retinol (also known as vitamin A).^38^ RA and its isomers such as all-*trans*-RA (ATRA), 9-*cis* RA, and 11-*cis RA*^39^ are involved in the regulation of different genes that are important for cell growth, differentiation, and apoptosis by activating the nuclear retinoid receptors, RA receptors (RARs) and retinoid X receptors (RXRs).^40^ The importance of ADH7 and RA in the adrenal gland has also been clearly shown in mammals. In mice, class IV ADH *(Adh4*, recently re-named *Adh7*, http://www.informatics.jax.org/searchtool/Search.do?query=adh&submit=Quick%0D%0ASearch) and RA are expressed in the embryonic adrenal glands and adult adrenal cortex.^41,42^ In fact, class IV ADH null mice are normal and fertile, with no growth defects when maintained on a normal diet. However, they show 100% lethality when placed on a vitamin A-deficient diet.^38,43^ These data suggest that *ADH7* is important in producing RA during development when vitamin A levels are low. Moreover, RA signalling may be involved in the regulation of immune cells. For example, reduction of primary murine B cell adhesion was observed after removing retinoids or by treatment with corticosteroids.^44^ These effects could suggest a putative RA-mediated activation of the immune system against ACC, a role that could be more important in paediatric and adult patients with and without Cushing syndrome.^45^

Although the effects of this SNP on RA signalling and ACC development remain to be further explored in mechanistic studies, we have been able to provide further evidence for the importance of the RA pathway genes in ACC. Specifically, using a two-step validation strategy to identify transcripts that significantly and reproducibly associate with ACC progression, we clearly demonstrated that transcripts for multiple RA pathway genes associate with differential progression in both adult (five genes) and paediatric (three genes) ACC. In both groups, we identified genes clearly involved in RA metabolism in a manner that could suggest that lower RA production associates with faster ACC progression. Specifically, in the adult ACC cohort, patients with low levels of *beta-carotene oxygenase 2* (*BCO2*) transcript associated with shorter PFI. *BCO2* is a mitochondrial enzyme involved in the production of retinoids from provitamin A carotenoids, which is a key source of retinoids in our diet.^46^ Specifically, BCO2 asymmetrically cleaves β-carotene to generate β-apo-10′-carotenal, which is then converted to retinoids by BCO1.^47,48^ In the paediatric ACC cohorts, shorter PFS was seen in patients with low transcript levels of *Aldehyde Dehydrogenase 1 Family Member A2* (*ALDH1A2*). ALDH1A2 is an enzyme involved in the rate-limiting step of RA production from retinaldehyde.^49,50^ Consistent with the hypothesis that less RA signalling results in faster ACC progression is the association of low mRNA levels for the *RA receptor beta* (*RARB*) with faster progression of adult ACC patients. RARB is a member of the RAR family that binds the retinoid ATRA to further activate down-stream RA signalling.^51^

This is not the first time that lower expression levels of the RA pathway genes have been implicated in ACC.^52^ In a meta-analysis of gene expression data from 164 adult adrenal tumours (97 adenomas and 67 carcinomas), significant reductions of three RA pathway transcripts were noted in malignant ACC relative to benign adrenal tumours. Specifically, *ALDH1A1, ALDH1A3, and RXRA* mRNAs were much lower in ACCs relative to adrenocortical adenoma.^52^ The authors, therefore, hypothesised that RA production and signalling might be reduced in ACC and, therefore, its re-activation could be a therapeutic option,^52^ as retinoids are already used in cancer treatment for acute promyelocytic leukaemia, and in cancer prevention for precancerous lesions in leukoplakia, actinic keratosis, and cervical dysplasia.^53^ Support of this came from experiments in both cell culture and murine xenografts, whereby treatment of ACC with 9-*cis* RA resulted in decreased cell viability, proliferation, and steroid hormone production.^54,55^ Our results offer human genetic evidence for the importance of this therapeutically relevant pathway in ACC onset and extends its relevance to progression of both paediatric and adult ACC. It is intriguing to note that a recent study of metastatic ACC tumours has identified an enrichment of driver mutations in RA pathway genes, possibly lending further support to the inactivation of this pathway being a crucial step in ACC progression.^56^ In addition, alterations in the retinoic acid pathway have also been linked recently to benign adrenocortical tumours secreting aldosterone.^57^

In conclusion, our results offer human genetic evidence for the importance of the druggable RA pathway in ACC onset and extend its relevance to progression of both paediatric and adult ACC. These findings will impact the design of future studies using RA in pre-clinical studies and guide preventive actions following genetic counselling and testing for children carriers of R337H germline mutation.

## Supplementary information

Surakhy et al, Supplementary data -final 2020

Supplementary data description

## Data Availability

The websites used in the analysis for this study are publicly available websites: http://www.cbioportal.org/, https://www.ebi.ac.uk/gwas, http://raggr.usc.edu http://hgdownload.soe.ucsc.edu/goldenPath/hg19/database/, https://www.ncbi.nlm.nih.gov/geo/query/acc.cgi?acc=%20GPL6801, http://software.broadinstitute.org/gsea/msigdb, https://www.ncbi.nlm.nih.gov/gds, http://firebrowse.org/. The data generated from the analysis during this study are included in this published article and its supplementary information files. Genotyping data for the Cancer Genome Atlas participants requires approval from the NIH.
