# Supplementary material for: A common polymorphism in the retinoic acid pathway modifies adrenocortical carcinoma age-dependent incidence
Source: Br J Cancer. 2020 Mar 9;122(8):1231–41. doi: 10.1038/s41416-020-0764-3 (PMC7156685; doi:10.1038/s41416-020-0764-3)
Supplement: Supplementary file 1 — Surakhy et al, Supplementary data -final 2020 [file 41416_2020_764_MOESM1_ESM.docx]

**A common polymorphism in the retinoic acid pathway modifies adrenocortical carcinoma age-dependent incidence**

Mirvat Surakhy^1^**^*^**, Marsha Wallace^1*^, Elisabeth Bond^1^, Lukasz Filip Grochola^2,3^, Husein Perez^4^, Matteo Di Giovannantonio^1^, Ping Zhang^1^, David Malkin^5^, Hannah Carter^6^, Ivy Zortea S. Parise^7^, Gerard Zambetti^8^, Heloisa Komechen^7^, Mariana M. Paraizo^7^, Meghana S. Pagadala^6^, Emilia M. Pinto^8^, Enzo Lalli^9^, Bonald C. Figueiredo^7,10#^, Gareth Bond^1^**^#^.**

|  |  |  |  |  |
| --- | --- | --- | --- | --- |

**Supplementary Fig. 1**

**
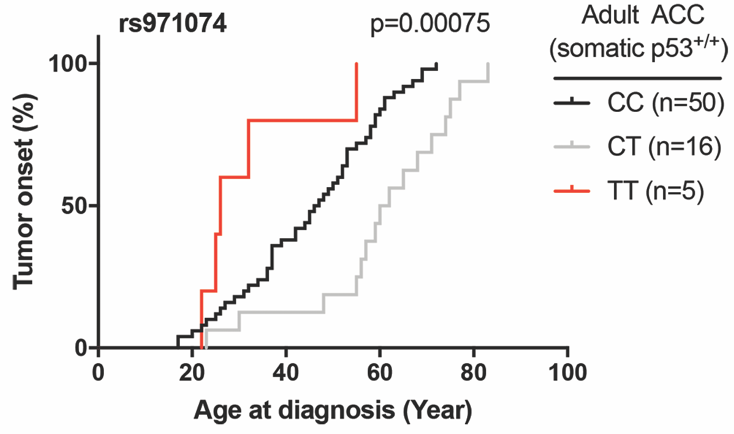
**

**Supplementary Fig. 1: rs971074 SNP modifies the age of ACC onset in *TP53* wild-type primarily adult patients.** Kaplan-Meier plot representing the age of tumor onset for both the major allele homozygous (CC), the heterozygous (TC) and the minor allele homozygous (TT) in the adult ACC cohort without somatic *TP53* mutations (n=71) (patients excluded: 18 patients have *TP53* somatic mutations, 2 have *TP53* germline mutation (R337H), and 1 patient the mutational status was not profiled), p=0.00075, Kruskal-Wallis test.

| **Supplementary Table 1.** Clinical features of adrenocortical carcinomas in pediatric patients carrying TP53 R337H mutation | | | | | | | | | | |  |
| --- | --- | --- | --- | --- | --- | --- | --- | --- | --- | --- | --- |
| **Patient Code** | **Age at diagnosis** | **Gender^a^** | **Clinical manifestation** | **Staging** | **Complete Surgery** | **QT/M^b^** | **R^c^** | **Outcome/ time** | **LFS^d^ or LFL^e^** | **Cohort** | |
|  |  |  |  |  |  |  |  |  |  |  |  |
| 1 | 1y^f^8m^g^ | F | V + C + H^h^ | I | Yes | No | No | well/35y | No | BRZ1 | |
| 2 | 1y | F | V | II | Yes | QT/M | No | well/10y | No | BRZ1 | |
| 3 | 1y | M | V | II | Yes | No | No | well/1y5m | No | BRZ1 | |
| 4 | 1y8m | F | V | I | Yes | No | No | well/9y | LFL | BRZ1 | |
| 5 | 2y | F | V | I | Yes | No | No | well/8y | No | BRZ1 | |
| 6 | 2y | F | V | I | Yes | No | No | well/10y | No | BRZ1 | |
| 7 | 2y | F | V + H | I | Yes | No | No | well/17y | LFL | BRZ1 | |
| 8 | 3y | F | V | I | Yes | No | No | well/8y | No | BRZ1 | |
| 9 | 3y | F | V | I | Yes | No | No | well/15y | LFL | BRZ1 | |
| 10 | 3y | M | V+C | II | Yes | QT/M | Yes | DD^i^ | No | BRZ1 | |
| 11 | 3y9m | F | NA^j^ | I | Yes | No | No | well/8y | LFL | BRZ1 | |
| 12 | 5y | F | V | I | Yes | No | No | No | No | BRZ1 | |
| 13 | 6y | F | V | III | Yes | QT/M | Yes | DD | LFL | BRZ1 | |
| 14 | 7y | F | V | II | Yes | No | No | well/7y | LFS | BRZ1 | |
| 15 | 7y | M | V | I | Yes | No | Yes | DD | LFL | BRZ1 | |
| 16 | 8m | F | V | I | Yes | No | No | well/7y | LFS | BRZ1 | |
| 17 | 9m | F | V | I | Yes | No | No | well/10y | LFL | BRZ1 | |
| 18 | 1y | M | V | I | Yes | No | No | well/20y | No | BRZ1 | |
| 19 | 1y1m | F | V | I | Yes | No | No | well/9y | No | BRZ1 | |
| 20 | 2y | F | V | I | Yes | No | No | well/9y | LFL | BRZ1 | |
| 21 | 2y | F | V | IV | Yes | QT/M | Yes | well/5y | LFL | BRZ1 | |
| 22 | 2y4m | F | V | I | Yes | No | No | well/8y | LFS | BRZ1 | |
| 23 | 4y4m | F | V | II | Yes | No | No | well/1y5m | No | BRZ1 | |
| 24 | 8m | M | V | I | Yes | No | No | well/10y | No | BRZ1 | |
| 25 | 10m | F | V + C + H | I | Yes | No | No | well/10y | LFL | BRZ1 | |
| 26 | 2y | F | V + C + H | I | Yes | No | No | well/9y | No | BRZ1 | |
| 27 | 5y1m | F | V | III | Yes | QT/M | No | well/9m | No | BRZ2 | |
| 28 | 1m22days | M | None | I | Yes | No | No | well/4y8m | No | BRZ2 | |
| \| Supplementary Table 1. continued \| \| --- \| | | | | | | | | | | | |
| 29 | 6y4m | M | V | III | Yes | QT/M | No | well/2y2m | LFL | BRZ2 | |
| 30 | 14y8m | M | V | IV | Yes | QT/M | No | well/1y | No | BRZ2 | |
| 31 | 1y6m | F | V | I | Yes | No | No | well/5y6m | LFL | BRZ2 | |
| 32 | 8y | F | V | III | Yes | QT/M | No | well/7y7m | LFL | BRZ2 | |
| 33 | 2y4m | M | V + C + H | IV | Yes | QT/M | No | well/9y | LFL | BRZ2 | |
| 34 | 9m | M | V | II | Yes | M | No | well/14y7m | No | BRZ2 | |
| 35 | 8m | F | V + C | I | Yes | No | No | well/10m | No | BRZ2 | |
| 36 | 1y | M | V | I | Yes | No | No | well/4y3m | No | BRZ2 | |
| 37 | 6m | F | V + C | III | Yes | QT/M | No | well/4y5m | LFL | BRZ2 | |
| 38 | 5y8m | M | V | III | Yes | QT/M | Yes | DD | No | BRZ2 | |
| 39 | 2y2m | M | V | II | Yes | M | Yes | well/8m | No | BRZ2 | |
| 40 | 1y9m | F | V | III | Yes | QT/M | No | well/8y | No | BRZ2 | |
| 41 | 1y2m | F | V + C | II | Yes | M | No | well/3y5m | LFL | BRZ2 | |
| 42 | 10m | F | V+C | IV | Partial | QT/M | No | well/with disease | LFL | BRZ2 | |
| ^a^Gender: F: female, M: male, ^b^QT/M: chemotherapy/mitotane, ^c^R: Recurrence, ^d^LFS: Li-Fraumeni syndrome, ^e^LFL: Li-Fraumeni like, ^f^y: year,  ^g^m: months, ^h^V: Virilizing syndrome, C: Cushing, H: hypertension, ^i^DD: died of disease, ^j^NA: not available | | | | | | | | | | |  |

| **Supplementary Table 2.** Clinical features of adrenocortical carcinoma in the primarily adult cohort | | | | | | | | | | | | | | | | |  | | |
| --- | --- | --- | --- | --- | --- | --- | --- | --- | --- | --- | --- | --- | --- | --- | --- | --- | --- | --- | --- |
| **Patient Code** | **Gender^a^** | **Race^b^** | **Days^c^** | **Treatment^d^** | **Stage** | **M** | **N** | **Age at diagnosis** | | **RM^e^** | | **R^f^** | **excess adrenal hormone^g^** | | | **p53 status** | |  |  |
| 1 | F | W | 2056 | Yes | I | M0 | N0 | 36 | No infil. | | | No |  | | | R335Lfs*10 | |  |  |
| 2 | F | W, H/L | 1352 | Yes | II | M0 | N0 | 22 | No infil. | | | Yes |  | | |  | |  |  |
| 3 | F | W | 562 | Yes | IV | M1 | N0 | 25 | Micro. | | | Yes | C | | |  | |  |  |
| 4 | F | W | 1194 | No | I | M0 | N0 | 26 | No infil. | | | No |  | | |  | |  |  |
| 5 | M | W | 2023 | Yes | II | M0 | N0 | 55 | No infil. | | | No |  | | |  | |  |  |
| 6 | F | W | 994 | Yes | III | M0 | N0 | 32 | No infil. | | | Yes | A, C | | |  | |  |  |
| 7 | F | W | 922 | Yes | IV | M1 | N0 | 53 | Micro. | | |  | A | | | E56*, X25_splice | |  |  |
| 8 | M | W | 579 | Yes | III | M0 | N0 | 66 | No infil. | | | Yes | C | | | T125_splice | |  |  |
| 9 | F | W | 3038 | No | II | M0 | N0 | 57 | No infil. | | | No |  | | |  | |  |  |
| 10 | F | W | 907 | Yes | II | M0 | N0 | 65 | No infil. | | |  | C | | |  | |  |  |
| 11 | F | W | 552 | Yes | IV | M1 | N1 | 68 | Macro. | | | Yes | A, C | | |  | |  |  |
| 12 | M | W | 822 | Yes | II | M0 | N0 | 60 | No infil. | | | Yes |  | | |  | |  |  |
| 13 | M | W | 3289 | Yes | II | M0 | N0 | 48 |  | | | Yes |  | | |  | |  |  |
| 14 | F | W | 1493 | Yes | II | M0 | N0 | 56 | No infil. | | | Yes | C | | |  | |  |  |
| 15 | F | W | 2076 | Yes | III | M0 | N1 | 55 | No infil. | | | No | A, C | | |  | |  |  |
| 16 | F | W | 1852 | No | II | M0 | N0 | 83 | No infil. | | | No |  | | |  | |  |  |
| 17 | F | W | 1317 | Yes | I | M0 | N0 | 77 | No infil. | | | No | E | | |  | |  |  |
| 18 | M | W | 1204 | Yes | IV | M1 | N0 | 59 | No infil. | | | Yes | A | | |  | |  |  |
| 19 | F | W | 159 | Yes | IV | M1 | N0 | 71 | Macro. | | | Yes | C | | |  | |  |  |
| 20 | F | W | 445 | No | III | M0 | N0 | 74 | No infil. | | | No |  | | |  | |  |  |
| 21 | M |  | 2740 | Yes | II | M0 | N0 | 62 | No infil. | | | No |  | | |  | |  |  |
| 22 | F |  | 1613 | Yes | II | M0 | N0 | 75 | No infil. | | | Yes | A, C | | |  | |  |  |
| 23 | M |  | 1858 | Yes | II | M0 | N0 | 23 | No infil. | | | No | E | | |  | |  |  |
| 24 | F |  | 853 | Yes | II | M0 | N0 | 30 | No infil. | | | No | M | | |  | |  |  |
| 25 | F | W | 383 | No | IV | M1 | N0 | 45 | Macro. | | | Yes | C | | | not profiled | |  |  |
| 26 | F | W | 741 | Yes | II | M0 | N0 | 61 | No infil. | | | Yes |  | | | V173L, K123N | |  |  |
| 27 | F | W, H/L | 1677 | Yes | IV | M1 | N0 | 44 | Macro. | | | Yes | A | | | H168Cfs*8 | |  |  |
| Supplementary Table 2. continued | | | | | | | | | | | | | | | | | |  |  |
| 28 | M | W, H/L | 365 | Yes | III | M0 | N0 | 30 | Macro. | | | Yes |  | | | R273C | |  |  |
| 29 | M | W | 551 | No | IV | M1 | N0 | 52 | Micro. | | |  |  | | | Y234C | |  |  |
| 30 | M | W | 541 | Yes | IV | M1 | N1 | 61 | Micro. | | |  | C | | | X125_splice | |  |  |
| 31 | M | W | 490 | Yes | IV | M1 | N1 | 65 | Macro. | | |  |  | | | C275S | |  |  |
| 32 | M | W | 464 | Yes | II | M0 | N0 | 40 | No infil. | | | No |  | | | E339Afs*8 | |  |  |
| 33 | F | W | 1414 | Yes | IV | M1 | N0 | 39 | No infil. | | | Yes | C | | | E339* | |  |  |
| 34 | F | W | 391 | Yes | IV | M1 | N1 | 23 | Macro. | | | Yes | A, C | | | C135Y | |  |  |
| 35 | M | W | 662 | Yes | II | M0 | N0 | 14 | No infil. | | | Yes |  | | | P2L7fs*17 | |  |  |
| 36 | F | W | 1105 | Yes | IV | M1 | N1 | 54 | Macro. | | | Yes | A, C | | | R213* | |  |  |
| 37 | M |  | 1293 | Yes |  |  |  | 63 |  | | | Yes |  | | | X307_splice | |  |  |
| 38 | F | A | 679 | Yes | III | M0 | N0 | 44 | Micro. | | | Yes | A, C | | | V73Rfs*76 | |  |  |
| 39 | F | W | 1103 | No | II | M0 | N0 | 26 | No infil. | | | No |  | | |  | |  |  |
| 40 | M | W | 2549 | No | II | M0 | N0 | 72 | No infil. | | | No |  | | |  | |  |  |
| 41 | M | W | 1355 | No | II | M0 | N0 | 58 | No infil. | | | Yes |  | | |  | |  |  |
| 42 | F | W, H/L | 1942 | Yes | III | M0 | N0 | 23 |  | | | Yes | A, C | | |  | | |  |
| 43 | F | W, H/L | 423 | Yes | IV | M1 | N1 | 23 | Macro. | | | Yes |  | | | Germline, R337H | | |  |
| 44 | F | B/Af M | 2703 | No | II | M0 | N0 | 29 | No infil. | | | No |  | | |  | | |  |
| 45 | F | W, H/L | 490 | Yes | III | M0 | N0 | 30 | Micro. | | | Yes | A, C | | | Germline, R337H | | |  |
| 46 | M |  | 1750 | No |  |  |  | 37 |  | | |  | E | | |  | |  |  |
| 47 | F | W | 2105 | Yes | I | M0 | N0 | 17 | No infil. | | | Yes | C | | |  | |  |  |
| 48 | F | W | 2015 | Yes | II | M0 | N0 | 69 | No infil. | | | No | C | | |  | |  |  |
| 49 | F | W | 2110 | No | II | M0 | N0 | 32 | No infil. | | | No |  | | |  | |  |  |
| 50 | M | W | 2331 | No | I | M0 | N0 | 22 | No infil. | | | No |  | | |  | |  |  |
| 51 | M | W | 1497 | Yes | IV | M1 | N0 | 49 | Macro. | | |  |  | | |  | |  |  |
| 52 | M | W | 3688 | No | I | M0 | N0 | 45 | No infil. | | | No |  | | |  | |  |  |
| 53 | F | W | 383 | Yes | II | M0 | N0 | 65 | No infil. | | | Yes |  | | |  | |  |  |
| 54 | F | W | 289 | Yes | III | M0 | N0 | 58 | No infil. | | | Yes |  | | |  | |  |  |
| 55 | M | W | 2202 | Yes | II | M0 | N0 | 47 | No infil. | | | No | C | | |  | |  |  |
| 56 | M | W | 950 | Yes | III | M0 | N0 | 50 | No infil. | | | No |  | | |  | |  |  |
| 57 | F | W | 1029 | No | II | M0 | N0 | 69 | No infil. | | | Yes | C | | |  | |  |  |
| 58 | M | W | 3465 | No | II | M0 | N0 | 53 | No infil. | | | No |  | | |  | |  |  |
| Supplementary Table 2. continued | | | | | | | | | | | | | | | | | |  |  |
| 59 | F | W | 1082 | Yes | III | M0 | N0 | 64 | Micro. | | | No |  | | | R337H | |  |  |
| 60 | F | W | 498 | Yes | III | M0 | N0 | 59 | No infil. | | | Yes | A, C | | |  | |  |  |
| 61 | M | W | 749 | Yes | II | M0 | N0 | 39 | No infil. | | | No | C | | |  | |  |  |
| 62 | F | W | 344 | Yes | II | M0 | N0 | 61 | No infil. | | | Yes | A, C | | |  | |  |  |
| 63 | F | W | 2777 | Yes | II | M0 | N0 | 45 | No infil. | | | Yes |  | | |  | |  |  |
| 64 | F | W | 2677 | No | II | M0 | N0 | 20 | No infil. | | | No |  | | |  | |  |  |
| 65 | F | W, Not H/L | 2895 | No | I | M0 | N0 | 44 | No infil. | | | Yes | A, C | | |  | |  |  |
| 66 | F | W | 4673 | Yes | II | M0 | N0 | 37 | No infil. | | | No |  | | |  | |  |  |
| 67 | F | W | 3878 | Yes | III | M0 | N1 | 17 | No infil. | | | Yes |  | | |  | |  |  |
| 68 | F | W | 1364 | Yes | III | M0 | N1 | 25 | No infil. | | | Yes |  | | |  | |  |  |
| 69 | M | W | 125 | Yes | II | M0 | N0 | 42 |  | | | Yes | C | | | E180_D184del | |  |  |
| 70 | F | W | 1781 | Yes | II | M0 | N0 | 37 | No infil. | | | Yes | C | | |  | |  |  |
| 71 | F | W | 4628 | No | I | M0 | N0 | 67 | No infil. | | | No | C | | |  | |  |  |
| 72 | F | W | 967 | Yes | III | M0 | N0 | 48 |  | | | No | A, C | | |  | |  |  |
| 73 | M | W | 861 | No | II | M0 | N0 | 60 | No infil. | | | No | A | | |  | |  |  |
| 74 | F | W | 885 | Yes | II | M0 | N0 | 36 | No infil. | | | No |  | | |  | |  |  |
| 75 | F | W | 871 | Yes | II | M0 | N0 | 53 | No infil. | | | No | A | | |  | |  |  |
| 76 | F | W | 719 | Yes | II | M0 | N0 | 52 | No infil. | | | No |  | | |  | |  |  |
| 77 | M | W | 1197 | Yes | III | M0 | N0 | 52 |  | | | Yes |  | | |  | |  |  |
| 78 | M | W | 1589 | Yes | III | M0 | N0 | 46 |  | | | Yes | C | | |  | |  |  |
| 79 | F | W | 2385 | Yes | II | M0 | N0 | 36 | No infil. | | |  | A | | |  | |  |  |
| 80 | F | W | 436 | Yes | IV | M1 | N0 | 42 | Macro. | | | Yes | A, C | | |  | |  |  |
| 81 | F |  | 2342 | No | II | M0 | N0 | 31 | No infil. | | | No |  | | |  | |  |  |
| 82 | F |  | 2405 | Yes | II | M0 | N0 | 61 | No infil. | | | Yes | A, E | | |  | |  |  |
| 83 | F |  | 1857 | No | II | M0 | N0 | 37 | No infil. | | | No |  | | |  | |  |  |
| 84 | F |  | 1096 | Yes | II | M0 | N0 | 34 | No infil. | | | No | A, C | | |  | |  |  |
| 85 | M |  | 549 | Yes | III | M0 | N0 | 57 | No infil. | | | No |  | | |  | |  |  |
| 86 | F | W | 709 | Yes | IV | M1 | N1 | 53 |  | | | Yes |  | | |  | |  |  |
| 87 | F | W | 207 | No | III | M0 | N0 | 55 |  | | | Yes | A, C | | |  | |  |  |
| Supplementary Table 2. continued | | | | | | | | | | | | | | | | | |  |  |
| 88 | F | W | 0 | No | IV | M1 | N0 | 59 | Macro. | | |  |  | | |  | |  |  |
| 89 | M | W | 756 | No | II | M0 | N0 | 51 | No infil. | | | No |  | | |  | |  |  |
| 90 | M | W | 3623 | No | II | M0 | N0 | 42 | No infil. | | | No |  | | |  | |  |  |
| 91 | F | A | 616 | No | II | M0 | N0 | 27 | No infil. | | | No |  | | |  | |  |  |
| 92 | M |  | 1201 | No | I | M0 | N0 | 63 | No infil. | | | No |  | | |  | |  |  |
| ^a^Gender: F: female, M: male. ^b^Race: W: White, H/L: Hispanic or Latino, A: Asian, B/Af M: Black or African American. | | | | | | | | | | | | | | | | |  | | |
| ^c^Days: days to last follow up or death. ^d^Treatment: chemotherapy and/or radiotherapy and/or mitotane. | | | | | | | | | | | | | |  | | |  | | |
| ^e^RM: surgical resection margins (No infil.: no infiltration, micro: microscopic infiltration, macro: macroscopic infiltration | | | | | | | | | | | | | | | | |  | | |
| ^f^R: recurrence, ^g^Excess adrenal hormone, C: cortisol, A: androgen, E: estrogen, M: mineralocorticoids | | | | | | | | | | | | | |  | | |  | | |
|  |  |  |  |  |  |  |  |  |  | |  | | | |  | |  | | |

| **Supplementary Table 3.** *TP53* mutations and the age at diagnosis of the  Canadian pediatric ACC patients | | | | |
| --- | --- | --- | --- | --- |
| **Patient Code** | **P53 mutation** | **Age at diagnosis**  **(yrs^a^)** | | **Gender^b^** |
| 1 | p.Cys275Ter | 3.67 | | M |
| 2 | p.His193Pro | 1.08 | | F |
| 3 | p.Phe134Tyr | 14.25 | | F |
| 4 | Deletion Exons 10-11 | 17.83 | | F |
| 5 | p.Glu180Lys | 2 | | M |
| 6 | p.Cys229Arg | 1 | | F |
| 7 | p.Gly334Arg | 1.75 | | F |
| 8 | p.Thr125Thr / splice | 1 | | F |
| 9 | p.Gln52fs | 15 | | F |
| 10 | p.Arg213Pro | 1 | | M |
| 11 | p.Ile254Thr | 2 | | F |
| 12 | p.Arg158His | 2.33 | | F |
| 13 | Pro152Leu | 2.5 | | M |
| 14 | p.Arg248Leu | 1 | | M |
| 15 | p.Thr125Thr / splice | 3 | | M |
| 16 | Deletion Exons 2-10 | 3 | | F |
| 17 | p.Cys229Arg | 13 | | F |
| 18 | splice | 4 | | M |
| 19 | p.Ser240Gly | 1.5 | | F |
| 20 | p.Cys229Arg | 5.5 | | F |
| 21 | p.Tyr163Cys | 2.67 | | F |
| ^a^yrs: year. ^b^Gender: F: female, M: male, | | |  |  |

| **Supplementary Table 4.** rs971074 in the Canadian pediatric ACC patients | | | | | |
| --- | --- | --- | --- | --- | --- |
| **Cohort** |  | | **Patient numbers** | **Mean (yrs^a^)** | **Median (yrs)** |
| **Canadian** | **Genotype** | Major (CC) | 14 | 5.01 | 2.59 |
|  |  | Minor (CT, TT) | 6, 1 | 4.14 | 2.33 |
|  | **MAF^b^** | | 0.1905 | | |
|  | **p-value (MWU^c^)** | | 0.9701 | | |
| ^a^yrs: years, ^b^MAF: Minor Allele Frequency^, c^MWU: Mann-Whitney *U* test | | | | | |

| **Supplementary Table 5:** RA pathway genes significantly associated with PFI in TCGA Discovery and Validation cohort pairs | | | | | | | | | | | | | | | | | | | | | | |
| --- | --- | --- | --- | --- | --- | --- | --- | --- | --- | --- | --- | --- | --- | --- | --- | --- | --- | --- | --- | --- | --- | --- |
|  | |  | | **Run number** | | | **1st** | **2nd** | | **3rd** | **4th** | **5th** | | **6th** | **7th** | | **8th** | **9th** | **10th** | | | **Percentage** |
|  | |  | | **Discovery (n, p <0.05)** | | | 20 | 28 | | 28 | 30 | 32 | | 30 | 27 | | 28 | 33 | 25 | | |  |
|  | |  | | **Validation (n, adjusted p<0.05)** | | | 9 | 7 | | 10 | 9 | 10 | | 7 | 8 | | 6 | 6 | 5 | | |  |
| **Gene** | | **RA gene classification** | | **Poor survival and mRNA level** | | |  |  | |  |  |  | |  |  | |  |  |  | | |  |
| **MAPK1** | | **Signal/Regu.** | | **high** | | | ✓ |  | | ✓ |  |  | |  |  | |  |  |  | | | 20% |
| **RARB** | | **Signal/Regu.** | | **low** | | | ✓ | ✓ | | ✓ | ✓ |  | |  | ✓ | | ✓ | ✓ |  | | | 70% |
| **CYP26B1** | | **Metabolic** | | **high** | | | ✓ | ✓ | | ✓ | ✓ |  | |  |  | |  | ✓ |  | | | 50% |
| **SCPEP1** | | **Signal/Regu.^a^** | | **low** | | | ✓ |  | | ✓ | ✓ | ✓ | | ✓ | ✓ | | ✓ | ✓ | ✓ | | | 90% |
| **BCO2** | | **Metabolic** | | **low** | | | ✓ |  | | ✓ |  | ✓ | | ✓ | ✓ | |  |  | ✓ | | | 60% |
| **CDK1** | | **Signal/Regu.** | | **high** | | | ✓ | ✓ | | ✓ | ✓ | ✓ | | ✓ |  | | ✓ | ✓ | ✓ | | | 90% |
| **PRKCA** | | **Signal/Regu.** | | **low** | | | ✓ | ✓ | |  | ✓ | ✓ | | ✓ | ✓ | | ✓ | ✓ | ✓ | | | 90% |
| **VDR** | | **Signal/Regu.** | | **high** | | | ✓ |  | |  |  |  | |  |  | |  |  |  | | | 10% |
| **UGT1A7** | | **Metabolic** | | **low** | | | ✓ | ✓ | |  | ✓ |  | | ✓ | ✓ | |  |  |  | | | 50% |
| **DHRS9** | | **Metabolic** | | **low** | | |  | ✓ | |  |  |  | |  |  | |  |  |  | | | 10% |
| **UGT1A9** | | **Metabolic** | | **low** | | |  | ✓ | | ✓ |  | ✓ | | ✓ | ✓ | |  |  |  | | | 50% |
| **CCNH** | | **Signal/Regu.** | | **low** | | |  |  | | ✓ |  | ✓ | |  |  | |  |  |  | | | 20% |
| **PRKCG** | | **Signal/Regu.** | | **high** | | |  |  | | ✓ | ✓ | ✓ | | ✓ | ✓ | |  |  |  | | | 50% |
| **MAPK14** | | **Signal/Regu.** | | **high** | | |  |  | | ✓ |  | ✓ | |  |  | | ✓ |  |  | | | 30% |
| **CYP1A1** | | **Metabolic** | | **high** | | |  |  | |  | ✓ |  | |  |  | |  |  |  | | | 10% |
| **RARA** | | **Signal/Regu.** | | **low** | | |  |  | |  | ✓ | ✓ | |  |  | |  |  |  | | | 20% |
| **CRABP2** | | **Metabolic** | | **high** | | |  |  | |  |  | ✓ | |  |  | |  |  | ✓ | | | 20% |
| **CYP26C1** | | **Metabolic** | | **high** | | |  |  | |  |  |  | |  | ✓ | |  |  |  | | | 10% |
| **CDK7** | | **Signal/Regu.** | | **low** | | |  |  | |  |  |  | |  |  | | ✓ | ✓ |  | | | 20% |
| Red: Genes that were associated significantly with PFI in at least 6 random pairs.  ^a^: Signal/Regu: Signaling /Regulation. | | | | | | | | | | | |  | |  |  | |  |  |  | | |  |
|  | |  | |  | | |  |  | |  |  |  | |  |  | |  |  |  | | |  |
|  | |  | |  | | |  |  | |  |  |  | |  |  | |  |  |  | | |  |
|  | |  | |  | | |  |  | |  |  |  | |  |  | |  |  |  | | |  |
| **Supplementary Table 6:** RA pathway genes associated with progression in pediatric ACC | | | | | | | | | | | | |  | | |  | | | | |  |  |
|  |  | |  | | Discovery | | | | Validation | | | | | | | | | | |  |  |  |
|  |  | |  | | IPACTR | | | | COG | | | | | | | | | | |  |  |  |
| Gene | descriptive gene name | | Transcript ID Array Design | | Probe Set ID | P-value | | | Probe Set ID | | | | P-value | | | adjusted P-value (Bonferroni) | | | | |  |  |
| ALDH1A2 | aldehyde dehydrogenase 1 family, member A2 | | g10835044 | | 207015_s_at | 0.00996844 | | | 207015_PM_s_at | | | | 0.0002786 | | | 0.0094728 | | | | |  |  |
| CDK1 | cyclin-dependent kinase 1 | | Hs.184572.0 | | 203213_at | 0.01474356 | | | 203213_PM_at | | | | 0.0004361 | | | 0.01482674 | | | | |  |  |
| PRKCA | protein kinase C, alpha | | Hs.80206.1 | | 213093_at | 0.00179756 | | | 213093_PM_at | | | | 2.56E-06 | | | 8.72E-05 | | | | |  |  |
| PRKCA | protein kinase C, alpha | | g4506066 | | 206923_at | 0.04787613 | | | 206923_PM_at | | | | 0.0001117 | | | 0.00379729 | | | | |  |  |
