# Supplementary material for: A common polymorphism in the retinoic acid pathway modifies adrenocortical carcinoma age-dependent incidence
Source: Br J Cancer. 2020 Mar 9;122(8):1231–41. doi: 10.1038/s41416-020-0764-3 (PMC7156685; doi:10.1038/s41416-020-0764-3)
Supplement: Supplementary file 2 — Supplementary data description [file 41416_2020_764_MOESM2_ESM.docx]

Supplementary data:

Figures

Supplementary Figure 1: plot for rs971074 SNP in *TP53* wild-type adult patients.

Tables

- - Supplementary Table 1 and 2: detailed clinical data of the Brazillian and adult ACC cohorts.
  - Supplementary Table 3 and 4: data of the Canadian pediatric ACC patients
  - Supplementary Table 5 and 6: association of the RA pathway genes with progression in pediatric ACC and adult ACC patients.
